# Post-2018 Ethiopia: state fragility, failure, or collapse?

**DOI:** 10.1057/s41599-022-01490-0

**Published:** 2022-12-22

**Authors:** Endalcachew Bayeh

**Affiliations:** Department of Political Science and International Studies, Bahir Dar University, Bahir Dar, Ethiopia

**Keywords:** Politics and international relations, Social policy

## Abstract

Owing to the multifaceted problems in which Ethiopia has been embroiled, such as war, displacement, and humanitarian crisis, writers arbitrarily use terms such as “state fragility,” “state failure,” and “state collapse” to represent the situation. This paper intends to determine what these concepts mean and to analyze whether post-2018 Ethiopia can be characterized by state fragility, failure, or collapse. To this end, I use a qualitative approach in which I gathered relevant data mainly from secondary sources. Accordingly, the study shows that state fragility, state failure, and state collapse are concepts related to the capacity and ability of the state to exercise a monopoly on the legitimate use of violence and perform its basic functions well. State fragility occurs if the state fails to exercise a monopoly on violence, protect its citizens, provide adequate public services, and maintain legitimacy. State failure, on the other hand, occurs when these problems become more critical, challenging the state’s existence. Finally, state collapse occurs when a state is completely disintegrated, leaving an authority vacuum. Based on this understanding, my findings indicate that Ethiopia is currently in a fragile condition and has started the process of descent into state failure. This apparent weakness of the state has devastating implications for the region, given the country’s previous stabilizing role, large population, and shared border with other regional states, among others. I argue that unless the necessary actions are taken, the situation might become worse and further destabilize an already volatile region.

## Introduction

State fragility, state failure, and state collapse have emerged as the main research agenda in various fields, including political science, since the end of the Cold War and the collapse of the Soviet Union. It has been common to see research reports on the issue, especially from Western development agencies and scholars. When dealing with this issue, researchers usually evaluate the capacity of the state to serve its purpose. It, therefore, is imperative that we know what a state is and what its functions are before we judge its performance and its fragility, failure, or collapse. While there is no consensus among scholars on the definition and functions of the state, and the concept has various definitions, I rely in this particular paper on Weber’s definition of the state as a human community that (successfully) claims a monopoly on the legitimate use of physical force within a given territory (Owen and Strong, 2004). An important element of this definition that is significant to determining the occurrence of state fragility, state failure, or state collapse is the monopoly on violence. The monopoly on violence is a prerogative of the state and is crucial to effectively performing its functions. The state’s quintessential function is to provide protection and security to its citizens. In addition, the state is supposed to provide certain basic services, including healthcare, education, and infrastructure, among others. These basic services vary among states based on their ideological orientation. For instance, many more services are expected from socialist and socially democratic states than from liberal states. State fragility, state failure, and state collapse, therefore, are conditions related to the state’s capacity to exercise its monopoly on violence and to provide security and basic services to its citizens, as shall be seen later.

The conditions of state fragility, state failure, and state collapse are especially common in African states that have followed an alien Western model of governance and nation-building, which has brought them unending ethnic conflict and political stability (Markakis, 2021). These conditions are especially prevalent in the Horn of Africa. “No state in this region has been able to adequately perform the functions generally assumed of it” (Markakis, 2021, p. 20). It is the most volatile region on the continent. It includes the first (Somalia) and the second (South Sudan) most fragile states in the world (The Fund for Peace, 2021). The case selected for this study is Ethiopia. This paper argues that despite an authoritarian nature and long-standing problems, the pre-2018 Ethiopian state had relatively better control over its territories and a monopoly on violence. Upon Prime Minister Abiy Ahmed’s accession to power in 2018, long-suppressed problems in the country resurfaced and have become uncontrolled. The state has faced serious governance challenges, such as interethnic conflicts, massive displacement, multiple massacres, enormous humanitarian crises, and the proliferation of militant groups. Consequently, it has been asserted that the state has already failed. This argument has been made, in particular, by Alex De Waal (2021b), one of the prominent scholars on the Horn of Africa. De Waal (2021a) argues that Abiy Ahmed’s “real authority extends no further than the first checkpoint outside the capital city.” Wondimu (2021) also writes, “Ethiopia is now on the brink of irreversible state collapse.” Whether these characterizations hold true is the focus of this study, which examines whether post-2018 Ethiopia is characterized by state fragility, state failure, or state collapse. No comprehensive study on this question has been conducted yet. The efforts made thus far have focused on merely describing the prevailing situation (Mosley, 2021; Donelli, 2022; Gov.UK, 2022; OCHA, 2022; Hassen and Rynn, 2022; Ethiopian Citizen, 2021; Hiiraan Online, 2022; BBC, 2021; AllAfrica, 2021). In addition, they have been predominantly media reports that represent diverse interests and, therefore, lack scientific rigor. In this study, therefore, I rely on a few synoptic definitions of state fragility, failure, and collapse and identify basic indicators that can be used as an analytical framework to understand the situation in Ethiopia. In doing so, the study is intended to address the following questions:

- What do state fragility, state failure, and state collapse mean?
- What does the situation in post-2018 Ethiopia look like?
- Which condition (fragility, failure, or collapse) characterizes post-2018 Ethiopia?

On the broadest level, by dealing with these questions, the study contributes to the existing body of knowledge in the area. This study adds to the discourse on state fragility, state failure, and state collapse and to the scant literature available on the Ethiopian case. Moreover, most studies conducted in this area have been quantitative, but this is purely qualitative, providing additional methodological contributions. As well as these contributions to knowledge and methodology, the study has policy implications for concerned bodies calling for actions to mitigate the situation and thus reduce its repercussions for the peace and stability of this already turbulent region.

## Methodology and methods

To address the above-stated questions, I have employed a qualitative methodology. Owing to the nature of the research questions, the method used is a descriptive study. And, the necessary data were collected from secondary sources such as books, journal articles, reports, and news. To substantiate the secondary data, I have also used my observations of what is happening.

## Conceptualization of terms

Like other social science concepts, state fragility, state failure, and state collapse have no commonly agreed upon single definition. These are highly politically motivated concepts. Western powers and development agencies use them to legitimize their intrusive interventions (Nay, 2013). They label states fragile, failed, or collapsed only when their interests are threatened (Nay, 2013). Hence, different actors suggest different definitions that are tailored to fit their respective interests. However, it should also be noted that weak states use fragility and failure to persuade the West/donors to continue their support (Clausen and Albrecht, 2022). This interest-driven understanding of the situation has resulted in many definitions and indicators of state fragility, failure, and collapse, making it difficult to study objectively. The following are the synoptic definitions I have relied on while developing the study’s conceptual framework.

According to McKay and Thorbecke (2016, pp. 3–4), a fragile state is “a low-income country characterized by weak state capacity and/or weak state legitimacy leaving citizens vulnerable to a range of shocks.” Similarly, Vallings and Moreno-Torres (2005, p. 4) argue that state fragility occurs because of “weak capacity and/or lack of political will to provide services and to sustain a development partnership with the international community.” Stewart and Brown (2009, p. 3) also define a fragile state as a state that fails to protect its citizens, provide public services, and have full legitimacy. As implied in these definitions and explicitly stated in The Fund for Peace (2021), monopoly over the legitimate use of force is also an important manifestation of fragility. These definitions show that fragility is a weakness in government institutions, which is analogous to what Rotberg (2003, p. 4) calls ‘weak states’ that do not provide an adequate level of public goods. In a nutshell, we can roughly say that a state is fragile if it fails to *monopolize violence*, *protect citizens*, provide adequate *social services*, and enjoy *legitimacy*.

According to Rotberg (2003, p. 5), “Failed states are tense, deeply conflicted, dangerous, and contested bitterly by warring factions.” For him, state failure occurs because of enduring civil wars of any kind. State failure is characterized by a lack of effective control of its territory, government attacks on its citizens, limited public services, a high prevalence of criminality, economic decline, deterioration of institutions and infrastructures, and a loss of legitimacy (Rotberg, 2003). These defining features of state failure significantly overlap with the aforementioned indicators of state fragility. Many researchers use state fragility and state failure interchangeably, but the condition of failure is a much more severe challenge to the continuity of the state as a state (Okeke et al., 2021). Woodward (2006) noted that states could be fragile, ineffective, abusive of human rights, and illegitimate without failing. Thus, failure is beyond mere weakness and ineffectiveness; instead, it “‘suggests a certain degree of finality’ where states are permanently subject to instability” (Pickering, 2014). Moreover, state power shrinks to the capital, losing control over sections of the territory and thereby failing to protect even central cities from the threat of rebel groups (Rotberg, 2003). The rolling back of state authority to the capital leaves citizens vulnerable, and thus, they begin to look for non-state security and social service providers (Rotberg, 2003). Therefore, state failure occurs when the above-stated fragility indicator problems deepen.

Unlike state fragility and state failure, according to Zartman (1995: 1), state collapse is “a situation where the structure, authority (legitimate power), law, and political order have fallen apart and must be reconstituted in some form, old or new.” Therefore, it is beyond mere weakness that may characterize fragility and failure. It is rather “a rare and extreme version of a state failure” (Rotberg, 2003, p. 9). These states are what Gros (1996) calls “anarchic states,” which do not have a central government. In conditions of state fragility and failure, the state may face various governance challenges, but the state still exists. In the case of state collapse, however, there is a complete disintegration of the state as a legitimate authority, thereby leaving an authority vacuum (Zartman, 1995; Rotberg, 2003). Thus, the state entirely ceases to function. Because of the absence of a government that provides security and public goods, “‘citizens’ are left to fend for themselves” (Emmanuel, 2012, p. 79). I am incorporating this phase (state collapse) not because I am assuming Ethiopia has reached this level, but to give conceptual clarity to the readers, as it is usually confused with state failure, and to show how near the country might be to this phase.

In a nutshell, for the sake of convenience, it is better to imagine that the three conditions lie in a continuum. They significantly overlap; thus, it is impossible to make a sharp demarcation between them, especially between fragility and failure. The only difference is the degree of the problem. If we take the arrow below, as one moves from fragility (the left end of the arrow) to collapse (the right end of the arrow), the degree of the problem increases.
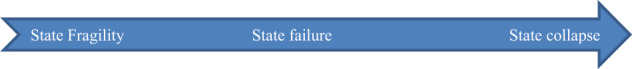


**Fig. 1:**
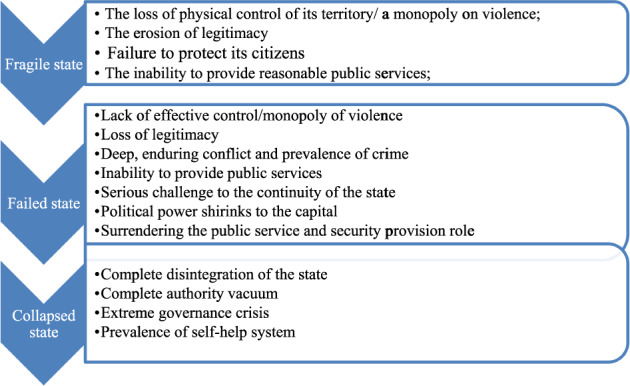
Concepts with their corresponding indicators. Source: Author’s own construction.

As it is difficult to come up with a cut point where a state becomes fragile or failed, I will rely on three basic questions, and if the findings show affirmative answers to them, then the country has failed; if not, it is fragile or is descending into failure. The following are the questions: Is the state’s authority or monopoly on violence limited to the capital? Are non-state actors and militant groups taking over the public service provision function of the state? Finally, are the local people resorting to non-state actors and militant groups for their protection or security? (Fig. 1).

## Pre-2018 Ethiopia: internal control and external role

Ethiopia is an old state that has never been colonized. It was thought to be a symbol of pan-Africanism that helped to decolonize African countries such as Zimbabwe and South Africa. It is a pivotal state for establishing the Organization of African Unity (OAU), now the African Union (AU). It hosts the headquarters. It was also among the first few countries to join the League of Nations, and later Ethiopia became a founding member of the United Nations (UN). Ethiopia also hosts the UN Economic Commission for Africa (ECA). It was an active participant in the maintenance of international peace and security. As part of the UN peacekeeping missions, its participation in the Korean and Congo wars cannot be left unmentioned. Ethiopia is also a linchpin of the HOA region. As an anchor state in the region, it has played a significant role in the attempts to bring peace and security to the regional states. In this regard, it is worth mentioning the role Ethiopia has played in efforts to resolve conflicts in Sudan, Somalia, and South Sudan both unilaterally and multilaterally via regional and international organizations. Generally, “Ethiopia is the largest troop contributor to UN peacekeeping with over 8,300 uniformed personnel, the vast majority of them serving in Darfur (UNAMID), Abyei (UNISFA), and South Sudan (UNMISS) (UN, n.d.).” Due to this external role, Ethiopia was labeled as the net exporter of stability and hope to the surrounding region (Verhoevena and Woldemariam, 2022).

Though the regime was characterized, inter alia, by authoritarianism, violation of human rights, and contested legitimacy, the state was relatively stable, with a solid military power capable of neutralizing internal and external threats (Mulugeta, 2016). It was a tightly controlled state capable of stifling the post-1991 intensification of not only ethnic mobilization but also contending nationalisms (Yusuf, 2019). Internal challenges, for instance, those from the Ogaden National Liberation Front (ONLF) and the Oromo Liberation Front (OLF), were not that strong (Yusuf, 2019). The EPRDF’s iron grip on power had managed to curtail internal armed insurgencies and silence interethnic violence. It was the only game in town. In addition to monopolizing violence, the regime also managed to achieve fast economic growth and significant reductions in maternal mortality, illiteracy, and HIV infections (Verhoevena and Woldemariam, 2022). In short, though the regime was authoritarian, it did not allow for lawlessness in its current form and the outburst of violence of any kind here and there. A few years before the accession to power of the current prime minister, protests began to intensify. A reform made in 2018 brought Abiy Ahmed to power and created widespread euphoria. Nonetheless, this soon relapsed and resulted in the prevailing turmoil in which the country has been embroiled. Now, citizens are in a state of fear, facing a lack of protection for their lives and property to a degree they have never experienced. Moreover, the inability of the state to enforce law and order and the tendency of citizens to rely on their own means of security have made Ethiopia’s domestic politics analogous to realist international politics (Yusuf, 2019). Therefore, the upcoming sections of the paper focus on the current situation in Ethiopia, especially following Prime Minister Abiy Ahmed’s assumption of office.

## The Post-2018 Ethiopia: what characterizes it?

### Greater optimism and immediate legitimacy crisis

One of the basic indicators of state fragility and failure is an erosion of legitimacy. A state is said to have faced a legitimacy crisis if there is an absence of a free and fair election, suppression of media and opposition, absence of popular support, and absence of civil and political liberties (Stewart and Brown, 2009). These signify the absence of democracy. Following Abiy Ahmed’s assumption of power, there was great optimism that the country would transition to a better system of administration where the citizens’ lives, liberty, property, and security were protected. Right after taking power, Abiy Ahmed introduced some remarkable legal and institutional changes and started liberalizing the political space. He released political prisoners, called up armed opposition parties operating abroad, lifted the state of emergency, and reformed the media and the Charities and Societies proclamations. Following this reform, various actors came to the political arena. These, together with the public discussions he conducted across the country, raised the hope and support of the people.

However, it did not take much time for the new prime minister to face a legitimacy crisis and for the people to lose their great optimism. The opening up of the political space has resurfaced the long-suppressed grievances of the people, raised their expectations, and unmasked interethnic tensions (Rameshshanker et al., 2020). The fact that Abiy could not manage these developments turned the people against his administration. Despite the progressive reforms, including freedom of expression, the new administration, like its predecessor, soon started arbitrarily arresting political opponents, activists, and journalists (Rameshshanker et al., 2020), thereby shrinking the political space. Though there are a plethora of media outlets, those expressing their dissent are being thrown into jail one by one, calling to mind what Idi Amin Dada once said: “there is freedom of speech, but I cannot guarantee freedom after speech” (Maureen, 2020, p. 4). The regime also jailed potential contenders on the eve of the national election and came out with a winner, which is an authoritarian culture that lingered from the previous regime. Besides, the regime has never hesitated to exclude from power those within the government cabinet who have a different opinion, i.e., what Abiy did to senior Amhara Prosperity Party members. It works only with those docile officials. This shows that the government has been preoccupied with consolidating its power at any cost. In some cases, its security apparatuses are being used to stifle dissent voices.

Political events in post-2018 Ethiopia are, to use Rameshshanker et al.’s (2020, p. 17) words, “isomorphic mimicry of a fully functioning democracy.” Therefore, owing to the oppressive and exclusionary acts of the government discussed above, “Ethiopia finds itself in a legitimacy trap” (Rameshshanker et al., 2020, p. 18). Popular support has dropped, and because of the intolerance of dissent voices and the inability to respond to various demands of the people, political and ethnic violence is spreading all over the country (Directorate for Sub-Saharan Africa, 2021; Yusuf, 2019). No part of the country is immune to violence, although this paper focuses on the major areas of prevailing political turmoil. The resultant inability of the government to provide protection and an adequate level of basic services to its citizens, as shall be seen below, further contributes to the deepening of its legitimacy crisis.

### Losing the monopoly on the legitimate use of violence

What makes a state a state, according to Max Weber, is its monopoly on the legitimate use of violence. The state should have complete control over the ownership, allocation, and movement of the means of violence. The absence of this confers the status of fragility or failure onto a state. Having this capacity is crucial in helping the state perform its functions properly. Nonetheless, this important state ingredient is somewhat currently missing in Ethiopia. Ethiopia has been embroiled in political turmoil, as evidenced by the crisis in the northern part of the country and insurgencies in other parts, notably the Oromia, Amhara, and Benishangul Gumuz regional states. Following the accession to power of Prime Minister Abiy Ahmed in 2018, the Tigray regional state has been at odds with the central government. It refused to join the Prosperity Party and conducted regional elections in 2020, rejecting the central government’s decision to postpone it (The Fund for Peace, 2021), thereby declaring its de facto independence. The tension turned into a full-blown war when the Ethiopian National Defense Force (ENDF) was attacked on November 4, 2020. Henceforth, Tigray has been entirely out of the hands of the government. Some areas of the Afar and Amhara regions had also come under the control of the Tigrayan forces. These developments demonstrate the weakening of the government in terms of controlling its territory. Quite recently, government forces have started liberating Afar and Amhara regions and pushing back Tigray People’s Liberation Front (TPLF) fighters. Despite this, the sustainability of such liberation and the rolling back of TPLF fighters remain fragile. However, the government lacks a monopoly on violence in the region. The war with Tigray significantly debilitated the national army, which, in turn, led to the proliferation of various militant groups in other parts of the country.

The Benishangul Gumuz region has also been the site of ethnic violence, which has claimed many lives and displaced many others. The region is one of the areas where the government has failed to govern properly. Repeated attacks have been made on ethnic Amhara and Agew people by criminal gangs (The Fund for Peace, 2021). Government officials are also targets of these ethnic Gumuz attacks. Moreover, the TPLF and the Oromo Liberation Army (OLA) have been accused of also operating in the region and taking part in the killing and displacement of the aforementioned ethnic groups and attacks on government officials (OCHA, 2022). As the Grand Ethiopian Renaissance Dam (GERD) is located in this region, it has been reported that Sudan and Egypt have supported the Gumuz militia (Donelli, 2022). Despite the government’s efforts to solve the recurrent hostilities in the region, the problem remains unabated. This also shows that the region is not under the complete control of the government. In short, the state does not have a monopoly on violence.

Moreover, in the Oromia region, there has been recurrent ethnic and political violence, causing the deaths and displacement of many people. Large areas of the region are under the control of the OLA, making government institutions in the area dysfunctional. Despite the legal registration of the OLF, its military wing, the OLA, has continued fighting against the federal government. It has a “conventional force with many new recruits and captured arms and munition” (Hassen and Rynn, 2022). They operate freely in the area. They have known camps, and they can organize people in a manner similar to government officials. In addition, they have killed government officials, looted local farms, and forcefully displaced other ethnic groups (Directorate for Sub-Saharan Africa, 2021). The Tigray conflict has allowed the OLA fighters to intensify their operations further after federal security agents left the area, leaving a power vacuum for the OLA to fill (Directorate for Sub-Saharan Africa, 2021). *Abbaa Torbee*, a clandestine organization, also operates in the area. The government has also accused it of bank robberies, targeted assassinations, and kidnappings (Directorate for Sub-Saharan Africa, 2021). Moreover, the *Qeerro*, a youth organization mobilized by Oromo political figures, has also engaged in alleged violent activities, including the expulsion of ethnic Amhara and Gurage (Directorate for Sub-Saharan Africa, 2021). Hence, the government has no complete control over the region, especially in the western, central, and southern Oromia regions (BBC, 2021). Eight of the 21 zones in the Oromia region are now under the control of OLA (Critical Threats, 2022).

The Amhara region is no exception. Because of long-standing oppression by the TPLF rule and the oppression of ethnic Amhara in other parts of the country, *Fano* has been operating outside the structure of the state (Critical Threats, 2022). Historically, the term “Fano” was used to refer to those who struggled against injustice and foreign invaders (Berhanu, 2022). Now, Fano is a youth Amhara militia fighting for the protection of the Amhara population (Berhanu, 2022). This force has been one of the key fighters on the central government’s side against the TPLF’s aggression. As the danger from, among others, TPLF persists, and some areas of the Amhara region remain under its control, Fano has continued training and preparing forces for the next possible war. Despite its role in the current war against the TPLF, the force remains outside the state apparatus. It is now considered a threat to the central government’s attempt to regain control over the region (Critical Threats, 2022), as seen from the government’s targeting of some of the group’s prominent figures. The fact remains that, here, too, the government could not monopolize the legitimate use of violence.

From the above discussion, we can deduce that the government has faced a challenge in exercising a monopoly over the legitimate use of force or in controlling the entire territories of the state. Also, the aforementioned instances show the fact that the state has started contracting and losing control over many parts of it. Considering the various militant groups operating in the country, it is possible to say, to use Ngunyi and Katumanga’s (2014) words, there is a shift from a monopoly of violence to an oligopoly of violence. However, even though the state’s authority is contracting, it is not as described by Alex De Waal (2021a) since most territories are under the government’s sovereign authority. Moreover, the expression of official power and the exercise of monopoly is not limited to the capital and a specific ethnic group, as is true with failed states (Rotberg, 2003).

### Failure to protect citizens and control criminal activities

The inability to provide security/protection to citizens and control criminal activities is another indicator of state fragility and failure. Providing security is the most crucial function that weighs very heavily in measuring state weakness and failure (Rotberg, 2003). Since Abiy’s government has faced a severe challenge in exercising a monopoly over the legitimate use of force, as discussed above, it has failed to properly serve its vital purpose, which is the security and protection of the people. Since the local government security apparatuses are weak in many parts of the country, especially those mentioned above, the local people are vulnerable and insecure. As discussed above, various militant groups commit various forms of violence, such as the OLA’s kidnapings, raids, displacements, and executions against civilians (Critical Threats, 2022), the despicable destruction that the TPLF has caused the Amhara and Afar region civilians and properties, the Gumuz criminal group attacks on ethnic Amharas and Agews, and the violence and robberies by armed individuals in the Amhara region. According to OCHA (2022), from April 2, 2018 to May 20, 2022, 2942 organized violence events and 16,091 total fatalities (of which 7270 were civilians) were reported. In addition, claims of ethnic cleansing and genocide are being made in various parts of the country, aggravating ethno-nationalist radicalization (Verhoevena and Woldemariam, 2022). Concerning this issue, the prime minister acknowledged the government’s failure to stop the continued alleged genocidal killings of civilians in his address to the parliamentarians, saying that “as a government, the fact we are not able to prevent the acts they committed, we feel quite sad” (Associated Press, 2022). As rightly put in Adinew and Abera (2021), these days, the perpetrators of such violations are primarily third parties, such as individuals, private groups, and other non-state entities, unlike in the past, when the state itself was the primary perpetrator. Therefore, we can boldly say that the government is neither properly respecting nor protecting citizens’ rights, security, and well-being.

There has been no redress for the injuries inflicted on the victims. People have lost trust in the government owing to its weakness in protecting and redressing them. Sizable people have more trust in local armed groups for their protection than the government forces. A case in point is the Amharas and Oromos. Because of the apparent failure of the government to protect the local Amharas and Amharas living elsewhere against multifaceted threats from various actors, sizable Amharas trust and rely on Fano rather than the government security forces for their protection. This shows some shift in allegiance from the state to local militant groups for protection, as evidenced by the recent popular protest against the government’s action on the Fano members. This is also true in volatile areas of the Oromia region, especially in western Oromia, where OLA has broader popular support (GOV. UK, 2022). Even incumbent party officials were blamed for clandestine joint work with it (Yusuf, 2019). This shows that, though not comparable with Somalia and South Sudan, which are notable examples of failed states where non-state actors are the basic security providers (Felbab-Brown, 2020; Iberi, 2021; Liaga, 2021), the people have started looking for non-state security providers.

The prevailing criminal activities and attendant militarization of society further exacerbated insecurity. Thanks to globalization and the ensuing widespread proliferation of Small Arms and Light Weapons (SALW), Ethiopian societies have become highly militarized to protect themselves. SALW has been smuggled across arbitrarily demarcated porous colonial boundaries of the region. This is true along Ethiopia’s border with Somalia, Kenya, South Sudan, and Sudan (Thrall and Cohen, 2022). It has been reported that Al-Shabaab poses a threat by smuggling arms into the poorly governed territories of the Somali region of Ethiopia (Hiiraan Online, 2022). Even it has recently carried out its “biggest-ever operation inside Ethiopia” (Maruf, 2022). This is partly due to the diversion of the government’s attention to the turmoil happening in the northern part of the country. The smuggling of arms in the peripheries, their penetration into the centers, and their existence in virtually everyone’s hands have worsened the insecurity.

**Fig. 2:**
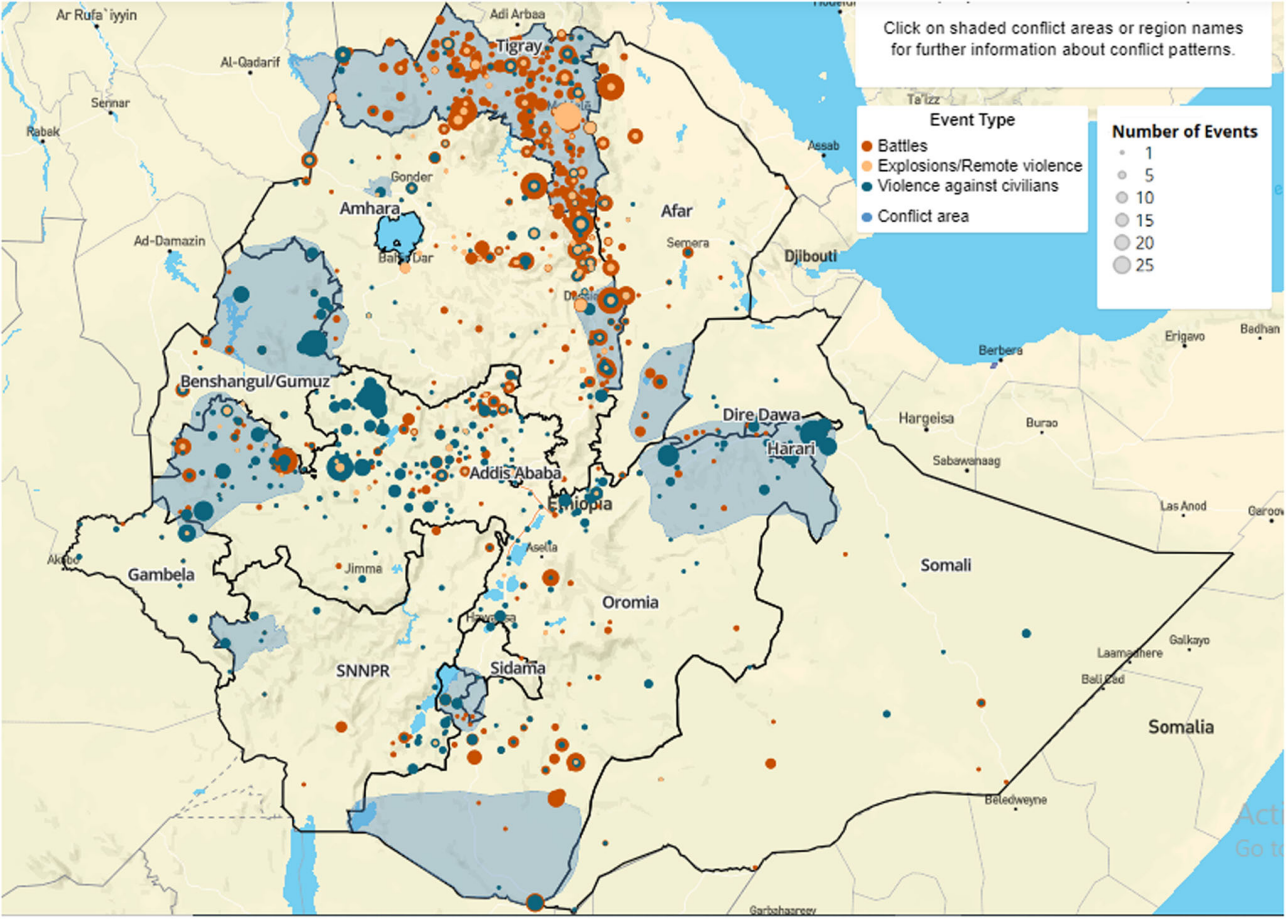
The picture shows organized political violence in Ethiopia (April 2, 2018–October 21, 2022). Source: Ethiopian Peace Observatory (2022).

To sum up, it seems that ensuring the security of the people remains challenging. Although the government is trying to create a platform for national dialog, I believe that peace remains fragile in Ethiopia given*,* inter alia, the deep polarization along ethnic lines, conflicting territorial claims of regional states, conflicting visions, conflicting historical narratives, and the massive material destruction and psychological trauma the conflicts have left. Hence, the prospect of this fragile government addressing incompatibilities, regaining a monopoly on the legitimate use of violence, and protecting citizens seem bleak, at least for the near future. Nevertheless, unlike failed states, despite the above-discussed deficiencies, the state remains the primary security provider (Fig. 2).

### Inadequate public service delivery

**Fig. 3:**
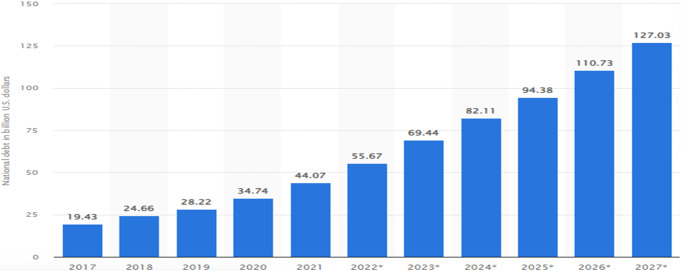
Ethiopian national debt from 2017 to 2027 (in billion U.S. dollars). Source: Aaron O’Neill (2022)*:* National debt of Ethiopia 2027.

The inability to provide public services is also another indicator of state fragility and failure. Public service involves basic education, health services, water, prevention of destitution, and infrastructure (Stewart and Brown, 2009). It also includes responding effectively to sudden shocks such as natural disasters, epidemics, food shortages, or refugee flows. A government provides adequate social services only when it has good economic performance. Because of the above-discussed and other problems, the Ethiopian economy is declining. Farmers are displaced from their areas, productive factories are burned, and investment enterprises are discouraged, all of which lead to an economic crisis, making the delivery of basic services difficult. For instance, Ethiopia’s free access to the American market via the framework of the African Growth and Opportunity Act (AGOA) has recently been suspended in relation to the war with Tigray, thereby adversely affecting the country and some previously exporting companies. In line with this, it has been reported that Best Garment, operating in Hawassa Industrial Park, alone has laid off more than 3000 workers because of market loss following restrictions from the AGOA (Mengesha, 2022). Ultimately, all the situations, such as the war the country is entangled in, COVID-19, the war in Ukraine, and drought, make the country’s economy show a dramatic downturn with a Real GDP in 2010–18 (9.7), 2019 (9.0), 2020 (6.1), 2021 (6.3) and projected to be 3.8 in 2022 (IMF, 2022, p. 28). This economic downturn not only debilitates the capacity of the state to provide basic services, as shall be seen below, but also further fuels political instability by, inter alia, involving the unemployed youth in violence. The country’s foreign debt burden has spiked due to this dramatic economic decline, as pictured below (Fig. 3).

Because of the armed struggle, social infrastructure and service delivery institutions are severely damaged in large areas. Schools, universities, hospitals, and roads, to mention just a few, are harshly ruined. It has been reported that 1600 schools teaching grades 1 to 12 were damaged, whereas 300 were completely destroyed (AllAfrica, 2021). This, in turn, made more than 1.2 million students out of school. Four universities in Tigray and one in Amhara regional state have been shut down, and students and teachers temporarily transferred to other universities (AllAfrica, 2021). In addition, in Afar and Amhara regions alone, more than three thousand health facilities have been destroyed (Ethiopian Citizen, 2021). These destructions and the aforementioned economic downturn have put the government in a difficult position to continue providing the expected services to its citizens in those affected areas. This is not to mention the inadequacy of services in areas that are relatively unaffected by violence as well.

Moreover, the country has encountered one of the world’s most severe humanitarian crises (Verhoevena and Woldemariam, 2022). The conflicts discussed above and the ensuing destruction, coupled with natural disasters, especially drought in the eastern and southern parts of the country, created a huge number of Internally Displaced People (IDPs) (over 4.2 Million (M)) and people desperately needing humanitarian support (~25.9M) (ACAPS, 2022). It is beyond its capacity to respond effectively to these sudden shocks. Addis Ababa alone receives more than 1000 refugees from affected regions per day (Critical Threats, 2022), which has made it the most expensive city in Africa (Benson, 2022). Generally, the living conditions of the people at large have highly deteriorated with a spectacular increase in the cost of living. “Hunger, ill health, skyrocketing housing, and crushing poverty” (God fly, 2021) are the order of the day.

This demonstrates the inability of the government to provide an adequate level of public services. Nonetheless, although inadequate and in some areas nonexistent, the state remains the primary service provider, unlike failed states where such a role is taken over by warlords and non-state actors (Rotberg, 2003), such as neighboring Somalia (Boogaard, 2021; Raballand and Knebelmann, 2021).

## Concluding remarks

Based on the findings discussed above, Ethiopia is a fragile state, as it exhibits the features of state fragility stipulated in the conceptual framework. As per the findings, we can also conclude that Ethiopia has started the process of descent into state failure as it has started manifesting some of the indicators of state failure mentioned in the conceptual framework. Though I do not agree with Alex De Waal, who argues that Ethiopia has already failed, it is undeniable that it has an apparent weakness in exercising a monopoly on violence, protecting citizens, providing public services, and securing legitimacy in many areas. However, it is difficult to say that it has reached what Pickering says is a “certain degree of finality” and faced what Okeke et al. say is a serious challenge to its continuity as a state. Moreover, though the state has started contracting, the expression of political power and monopoly of violence does not shrink to the capital as it happens in failed states. The state still exercises authority over a large part of its territory and remains the primary security and social service provider. Thus, it is a bit early to conclude that the state has failed. However, it is fair to say that it has started the process of descent into state failure. Obviously, it is not a collapsed state. No matter how weak and undemocratic it is, there is an elected government, which is more or less performing its functions in a large part of the country. However, if the current situation continues anymore, there is no reason why it will not fail like Somalia and South Sudan and descend into the worst case of failure, which is state collapse. Given the long-standing remarkable history of survival and statehood resilience, the country can be salvaged from the condition of complete failure. This, however, needs a concerted effort from all concerned bodies. Otherwise, not just Ethiopia but the entire region will be destabilized given, inter alia, the country’s previous stabilizing role in the region, large population, and border share with many of the regional states.

## Data Availability

All data analyzed are contained in the paper.
